# Identification of triptonide as a therapeutic agent for triple negative breast cancer treatment

**DOI:** 10.1038/s41598-021-82128-0

**Published:** 2021-01-28

**Authors:** Bowen Gao, Jiongyu Chen, Bingchen Han, Xinfeng Zhang, Jijun Hao, Armando E. Giuliano, Yukun Cui, Xiaojiang Cui

**Affiliations:** 1 Department of Surgery, Samuel Oschin Comprehensive Cancer Institute, Cedars-Sinai Medical Center, 8700 Beverly Blvd, Davis Building 2065, Los Angeles, CA 90048 USA; 2 Guangdong Key Laboratory for Breast Cancer Diagnosis and Treatment, Cancer Hospital of Shantou University Medical College, Shantou, 515041 China; 3 College of Veterinary Medicine, Western University of Health Sciences, Pomona, CA 91766 USA

**Keywords:** Breast cancer, Cancer therapy

## Abstract

Triple-negative breast cancer (TNBC) is associated with a high rate of early recurrence and distant metastasis, frequent development of therapeutic resistance, and a poor prognosis. There is a lack of targeted therapies for this aggressive subtype of breast cancer. Identifying novel effective treatment modalities for TNBC remains an urgent and unmet clinical need. In this study, we investigated the anti-cancer effect of triptonide, a natural compound derived from the traditional Chinese medicinal herb *Tripterygium wilfordii* Hook F, in TNBC. We found that triptonide inhibits human TNBC cell growth in vitro and growth of TNBC xenograft mammary tumors. It induces apoptosis and suppresses stem-like properties as indicated by reduced mammosphere formation and aldehyde dehydrogenase activity in TNBC cells. We show that triptonide downregulates multiple cancer stem cell-associated genes but upregulates SNAI1 gene expression. In support of SNAI1 induction as a negative feedback response to triptonide treatment, in vitro-derived triptonide-resistant HCC1806 cells display a markedly higher expression of SNAI1 compared with parental cells. Mechanistically, the increase of SNAI1 expression is mediated by the activation of JNK signaling, but not by ERK and AKT, two well-established SNAI1 regulators. Furthermore, knockdown of SNAI1 in the triptonide-resistant HCC1806 cells increases sensitivity to triptonide and reduces mammosphere formation. These results indicate that triptonide holds promise as a novel anti-tumor agent for TNBC treatment. Our study also reveals a SNAI1-associated feedback mechanism which may lead to acquired resistance to triptonide.

## Introduction

Triple-negative breast cancer (TNBC) is defined by the lack expression of estrogen receptor (ER), progesterone receptor (PR), and human epidermal growth factor receptor 2 (HER2)^1^. TNBC is associated with a poor prognosis and high rate of early recurrence and distant metastasis. Chemotherapy is the only common systemic treatment for TNBC patients, although PD-L1 immunotherapy has been approved for a small subset of those patients^2^. TNBC is heterogeneous in gene expression and genomic features, further complicating the task to develop targeted therapies to treat this aggressive subtype of breast cancer. Although advances in breast cancer treatment have taken a quantum leap in recent years, novel effective therapeutics for TNBC remains a clinically unmet need.

Triptonide (TN) and triptolide (TL) are two major bioactive chemical compounds identified in *Tripterygium wilfordii* Hook F, which has historically been used in traditional Chinese medicine to treat rheumatoid arthritis for centuries^3^. Both compounds have been known to have a broad spectrum of biological functions such as immunosuppression, anti-inflammation, anti-fertility, and neuroprotective effects^4^. Particularly, the anticancer activity of triptolide has attracted the most interest and recently has been investigated in different cancers. Of note, *Saluja* et al. have synthesized analogs of triptolide that are able to overcome oxaliplatin resistance by downregulating the DNA Repair Pathway in pancreatic cancer^5^. *Yang* et al. have reported that triptolide selectively deplete cancer stem cells (CSCs) while sparing normal tissue stem cells in human TNBC cell lines^6^. However, the adverse reactions of triptolide, including hepatotoxicity and nephrotoxicity, have been frequently reported^7^. Therefore, those severe toxicities may limit the therapeutic potentials of triptolide in cancer treatment.

**Figure 1:**
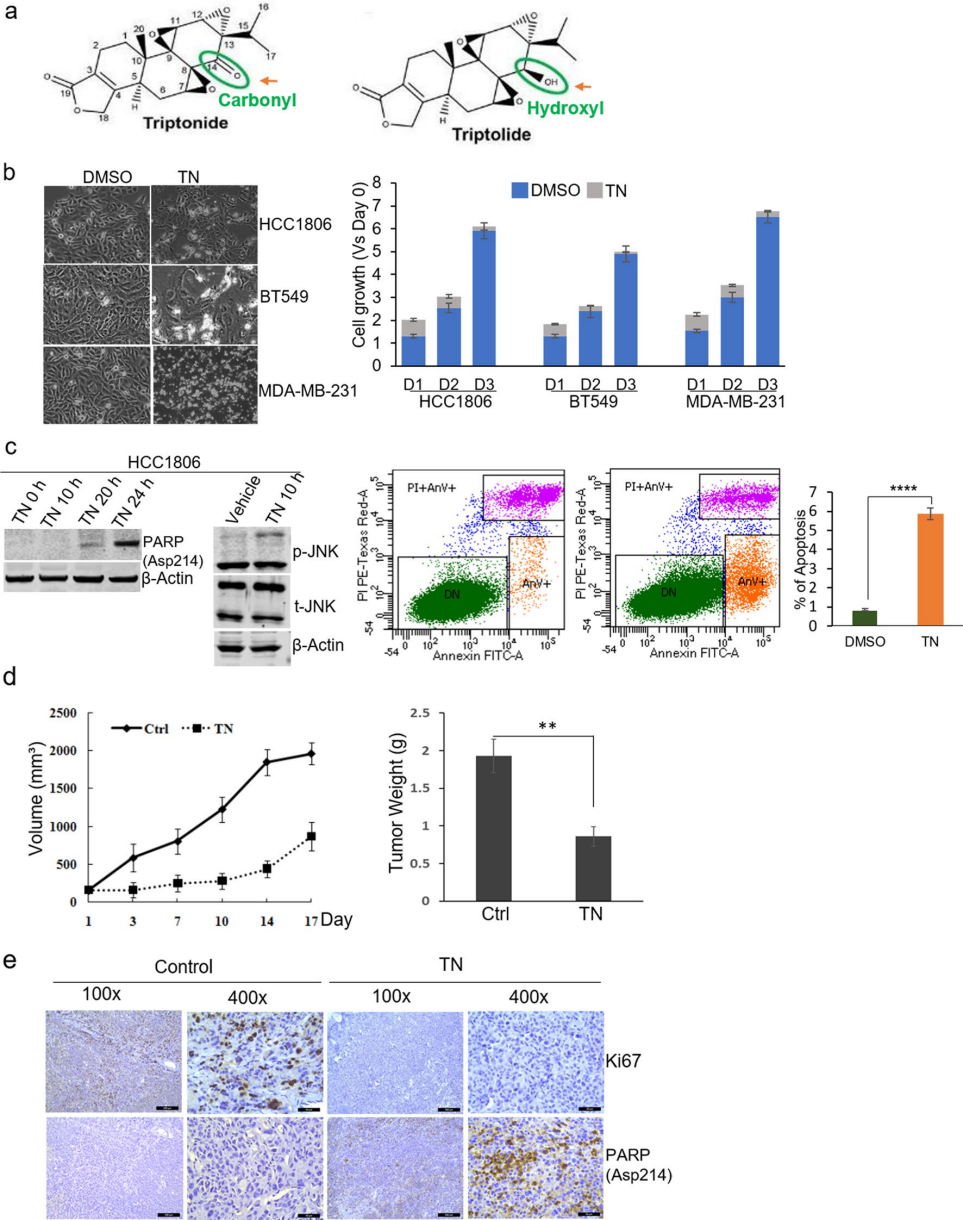
Triptonide inhibits TNBC cell growth in vitro and in vivo. (**a**) Chemical structures of triptonide and triptolide. (**b**) Left, representative images showing the effects of 0.2 µM triptonide treatment for 24-h in HCC1806, BT549, and MDA-MB-231 cells. Magnification, × 200. Right, quantified effects of 0.2 µM triptonide on cell growth measured by CellTiter-Glo assays. HCC1806, BT549, and MDA-MB-231 cells were treated for 24 h (D1), 48 h (D2) and 72 h (D3). Each bar is presented as mean ± SD (n = 3). (**c**) Left, immunoblotting of p-JNK, total (t)-JNK, cleaved PARP in HCC1806 cells treated with 0.2 µM triptonide. Right, apoptosis analysis of HCC1806 cells treated with 0.2 µM triptonide for 20 h by the FITC Annexin V/PI apoptosis assay. (**d**) Comparisons of MDA-MB-231 xenograft mammary tumors in mice treated with vehicle or triptonide. Graphs show tumor volume changes with time and tumor weight at harvest. Each bar is presented as mean ± SD (n = 4). **, p < 0.01. (**e**) Expression of Ki67 and cleaved PARP in MDA-MB-231 xenograft tumor tissues examined by immunohistochemistry assays. Magnification, × 400.

Triptonide molecule has a similar structure with triptolide but only differs by having a carbonyl chemical group at C position 14, in which triptolide has a hydroxyl chemical group (Fig. 1a). This structural difference is associated with the markedly lower toxicity of triptonide in vivo when compared with triptolide^8^. The lower toxicity of triptonide with potent antitumor activity has been getting much attention. One recent study demonstrated that triptonide effectively inhibits Wnt/β-catenin signaling by targeting the downstream C-terminal transcription domain of β-catenin in STF293 cells^4^. Triptonide has also been shown to exert a potent anti-leukemia effect through the induction of leukemia cell senescence^9^. inhibits the growth of gastric cancer-associated fibroblasts and prostate cancer through mTOR signaling pathway^10,11^. and suppresses pancreatic cancer cell-mediated vasculogenic mimicry by inhibiting expression of VE-cadherin^12^. To date, the effects of triptonide on breast cancer, including TNBC, have not been reported.

To explore the potential use of triptonide in breast cancer treatment, the present study was designed to test the effect of triptonide on TNBC cells growth and stem cell properties and delineate the potential mechanisms of resistance to the treatment. For this purpose, we conducted cell viability assays, mammosphere formation assays, RT^2^ profiler PCR array analysis, breast cancer xenograft studies, and established a triptonide-resistant TNBC cell line. We found that triptonide exerts a potent inhibitory effect on TNBC cell growth in vitro and in vivo. The expression of CSC-associated genes was also regulated by triptonide. Expression of SNAI1, a well-known transcription factor involved in the epithelial mesenchymal transition (EMT), was markedly induced in triptonide-resistant TNBC cells. As expected, knockdown of SNAI1 restored cell sensitivity to triptonide. Mechanistically, JNK activation was found to mediated triptonide-induced SNAI1 expression. These results suggest a potential cell response counteracting the apoptosis-inducing effects of triptonide. Elucidating the underlying feedback mechanism may help develop and optimize triptonide-based alternative and complementary therapy for TNBC.

## Results

### Triptonide inhibits TNBC cell growth in vitro and in vivo

We first used the human TNBC cell line HCC1806 to examine the effects of different triptonide concentrations on cell growth in vitro. The dose–response assay with a 24-h time point of triptonide treatment showed that maximal. We then conducted time-course experiments by treating HCC1806 cells with 0.2 µM triptonide. As presented in Fig. 1b, the reduction of cell growth by triptonide continued with time, whereas the growth of the vehicle control increased over the same time period. Similar results of the effect of triptonide on tumor cell growth were observed in two other common human TNBC cell lines BT549 and MDA-MB-231 (Fig. 1b) as well as the ER + cell line MCF7 and the HER2 + cell line SKBR3 (Fig. S1b). In support of these results, immunoblotting showed that cleaved poly (ADP-ribose) polymerase (PARP), a marker of cell apoptosis, was induced by triptonide in TNBC cells (Fig. 1c). The c-Jun N-terminal kinase (JNK), which is well-established for its essential role in anti-cancer agent-elicited apoptosis, was also activated by triptonide as indicated by immunoblotting of increased phosphorylated JNK (p-JNK) (Fig. 1c). Furthermore, the FITC-annexin V/Propidium Iodide (PI) apoptosis assay confirmed the apoptosis-inducing effect of triptonide in TNBC cells (Fig. 1c, right panel).

Next, we investigated the in vivo effect of triptonide on TNBC cell growth using the MDA-MB-231 xenograft model. To do so, MDA-MB-231 cells were orthotopically injected into the mammary fat pad of female nude mice. As shown in Fig. 1d, compared with the control group, triptonide treatment significantly reduced the growth of xenograft mammary tumors. The volume and weight of tumors in the triptonide treatment group were markedly lower than those in the control group at the end of treatment. Furthermore, immunohistochemistry analysis showed that expression of the cell proliferation marker Ki67 was decreased, while cleaved PARP expression was increased, in the tumors treated by triptonide (Fig. 1e). Apoptosis induction by triptonide in xenograft tumors was also revealed by TUNEL assays (Fig. S1c). Taken together, these data indicate that triptonide is a potent inhibitor of TNBC cell growth.

### Triptonide attenuates stem-like properties of TNBC cells

**Figure 2:**
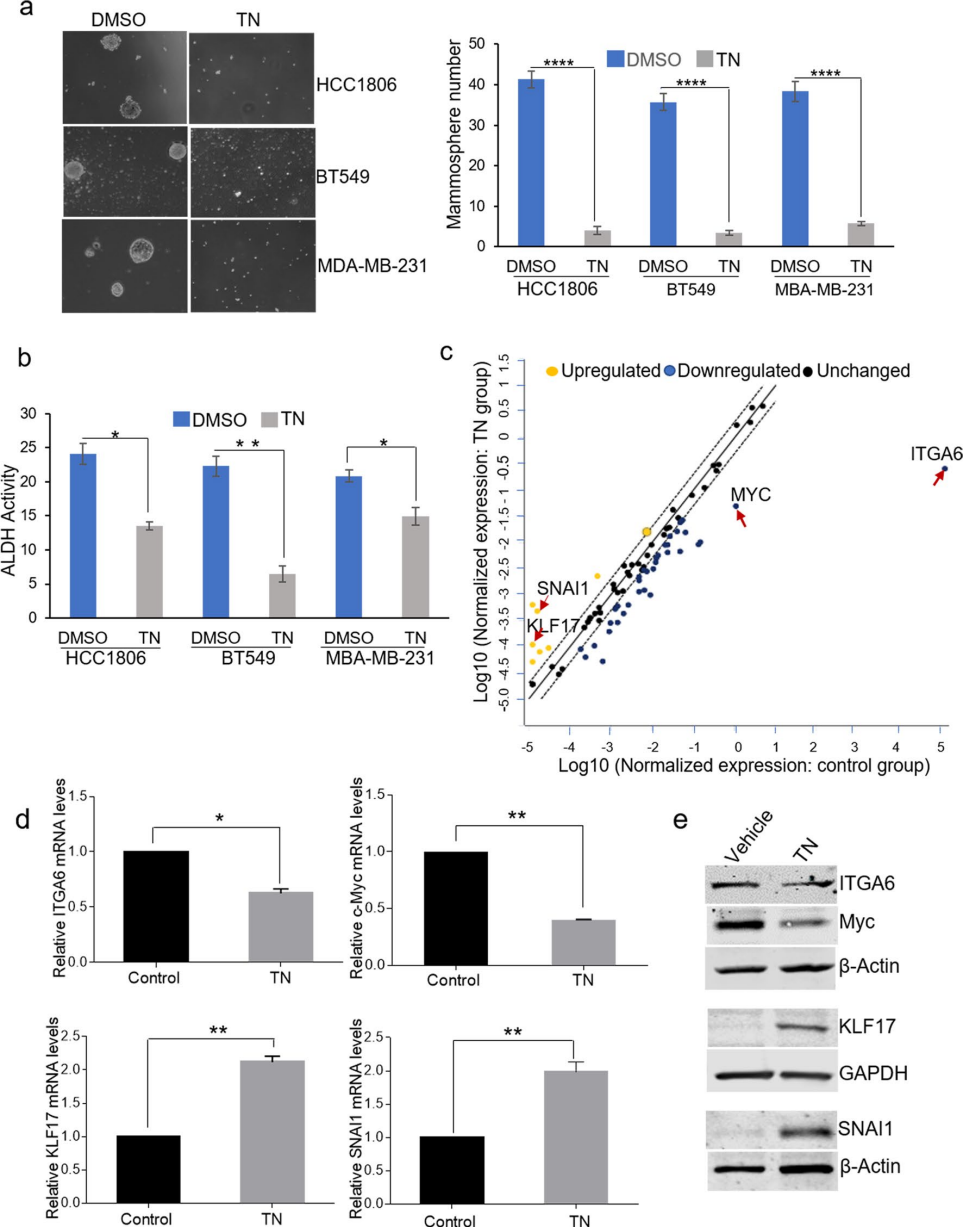
Triptonide attenuates stem-like properties of TNBC cells. (**a**) Mammosphere formation assay in HCC1806, BT549, and MDA-MB-231 TNBC cells treated by triptonide for 96 h. Each bar is presented as mean ± SD (n = 3). (**b**) ALDH activity assays of HCC1806, BT549, and MDA-MB-231 cells treated by triptonide or vehicle for 20 h. Each bar is presented as mean ± SD (n = 3) (**, p < 0.01). (**c**) RT^2^ profiler PCR array of HCC1806 cells treated with 0.2 µM triptonide for 10 h using the ΔΔCT method of quantification with RT2 Profiler PCR Arrays kit. (**d**) Expression of ITGA6, Myc, KLF17, and Snail by RT-qPCR. Each bar is presented as mean ± SD (n = 3) (*, p < 0.05. **, p < 0.01). (**e**) Immunoblotting of ITGA6, MYC, KLF17, and SNAI1 in HCC1806 cells.

Given that cancer stem cells (CSCs) play a critical role in cancer initiation, metastasis, and drug resistance^6^, we examined whether triptonide exerts effects on CSC properties. To this end, mammosphere formation assays were performed. As shown in Fig. 2a, the numbers of mammospheres in HCC1806, BT549, and MDA-MB-231 cells were decreased drastically by triptonide treatment compared with the vehicle control. Using aldehyde dehydrogenase (ALDH) activity colorimetric assays, we observed that ALDH activity, another common biomarker for characterizing CSCs, was also substantially reduced by 20-h triptonide treatment in HCC1806, BT549, and MDA-MB-231cells (Fig. 2b). To assess whether triptonide alters stem cell gene expression, we conducted Human Stem Cell RT^2^ Profiler PCR Array analysis of HCC1806 cells (Fig. 2c). Among the 84 genes related to stem cell identity, function, and differentiation contained in the array, 14 genes including *ITGA6* and *MYC* were downregulated and 6 genes including *KLF17* and *SNAI1* were upregulated by triptonide treatment for 10 h with at least a fourfold change (Supplementary Fig. S2a). To validate the array results, we conducted real-time reverse transcription PCR (RT-qPCR) analysis of the expression changes of *ITGA6, MYC, KLF17, and SNAI1* in HCC1806 cells (Fig. 2d) and MDA-MB-231cells (Supplementary Fig. S2b). Immunoblotting further confirmed that the protein levels of MYC and ITGA6 were decreased while those of KLF17 and SNAI1 were increased by triptonide treatment (Fig. 2e). Collectively, these data suggest that triptonide diminishes CSC properties and alters CSC gene expression in TNBC cells.

### SNAI1 induction is a feedback response to triptonide treatment

**Figure 3:**
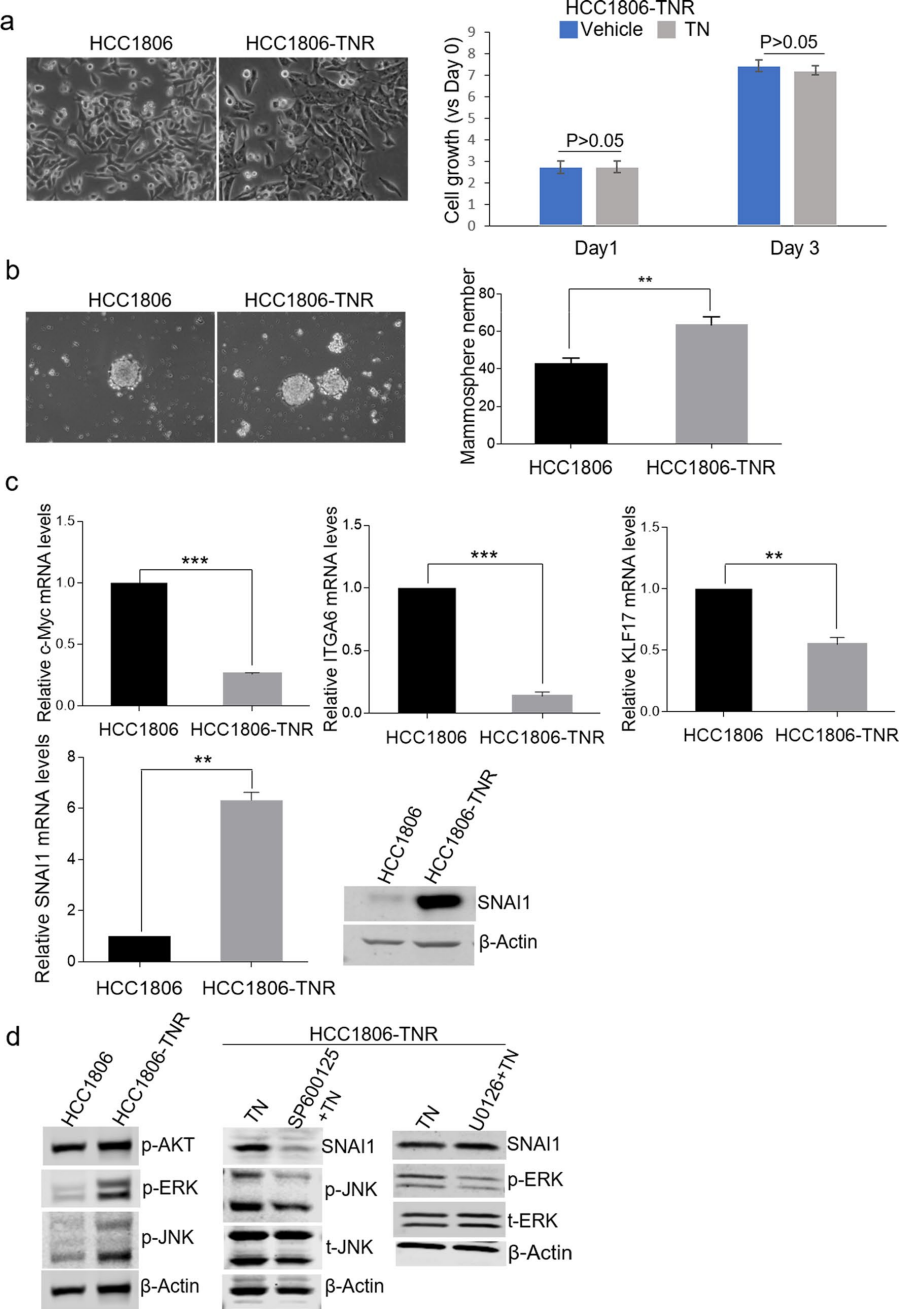
SNAI1 expression is induced in TN-resistant HCC1806 cells. (**a**) Comparisons of parental and TN-resistant HCC1806 cell morphology and growth rate in TN-resistant HCC1806 cells with or without triptonide treatment. Each bar is presented as mean ± SD (n = 3). Magnification, × 200. (**b**) Mammosphere formation ability of TN-resistant HCC1806 cells compared with parental cells. (**, p < 0.01). (**c**) mRNA expression of ITGA6, Myc, KLF17, and SNAI1 in TN-resistant HCC1806 cells compared with parental cells. Each bar is presented as mean ± SD (n = 3) (*, p < 0.05. **, p < 0.01, ***, p < 0.001). SNAI1 protein levels were also measured by immunoblotting. (**d**) Immunoblotting of p-AKT, p-ERK, p-JNK (left panel) in parental and TN-resistant HCC1806 cells. Immunoblotting of SNAI1 and p-JNK or p-ERK in TN-resistant HCC1806 cells treated with the JNK inhibitor SP600126 (10 µM) and the ERK inhibitor U0126 (10 µM).

Downregulation of MYC and ITGA6 and upregulation of KLF17, a negative regulator of the epithelial-mesenchymal transition (EMT) and metastasis in breast cancer^13^, are consistent with the anti-tumor effects of triptonide in TNBC cells. However, the increase of SNAI1 expression appears to be paradoxical, as it induces CSC traits and resistance to apoptotic stimuli^14^. Thus, we reasoned that the SNAI1 increase in treated-TNBC cells may represent a negative feedback response to triptonide. Given that cellular feedback mechanisms can eventually lead to drug resistance in cancer treatment, we proceeded to explore the role of SNAI1 in the development of resistance to triptonide treatment in TNBC. For this purpose, we generated a triptonide-resistant (TN-resistant) HCC1806 sub-line by continuously growing parental HCC1806 cells with increasing concentrations of the agent until cultured cells showed no growth reduction in the presence of 0.2 µM triptonide. As illustrated in Fig. 3a, the morphology of TN-resistant HCC1806 cells was similar to that of parental cells (left panel) but the growth of this sub-line was not inhibited by triptonide (right panel). Compared with parent cells, these TN-resistant cells exhibited an enhanced mammosphere formation capacity (Fig. 3b). RT-qPCR analysis showed that the levels of Myc, ITGA6, and KLF17 mRNA were lower in these cells than those in parental cells (Fig. 3c). In contrast, SNAI1 mRNA expression was markedly elevated in these TN-resistant cells, which was also in line with the SNAI1 immunoblotting results (Fig. 3c).

Because AKT and ERK are well-established activators of SNAI1 expression in cancer cells^15,16^, we tested whether they regulate the drastic elevation of SNAI1 levels in TN-resistant TNBC cells. To do so, we compared activated AKT and ERK levels in TN-resistant and parental cells using immunoblotting. As shown in Fig. 3d, phosphorylated EKT (p-ERK) levels were increased in TN-resistant cells, while p-AKT levels did not change. Consistent with our finding of p-JNK induction by triptonide in TNBC cells, p-JNK levels were also found to be higher in TN-resistant cells, which are routinely cultured in the presence of triptonide. Surprisingly, inhibition of ERK using a specific small-molecule inhibitor U0126 did not affect SNAI1 expression as indicated by immunoblotting (Fig. 3d, right panel), but inhibition of JNK using its inhibitor SP600125 diminished SNAI1 levels. The differential effects of ERK and JNK on SNAI1 expression were confirmed in parental HCC1806 cells treated by triptonide (Fig. S3). Of note, few reported studies have shown that JNK regulates SNAI1 expression in cancer cells. Taken together, these data suggest a SNAI1-mediated negative feedback response to triptonide and a role of JNK activation in SNAI1 induction in TNBC cells.

### SANI1 is essential for acquired resistance to triptonide in TNBC cells

**Figure 4:**
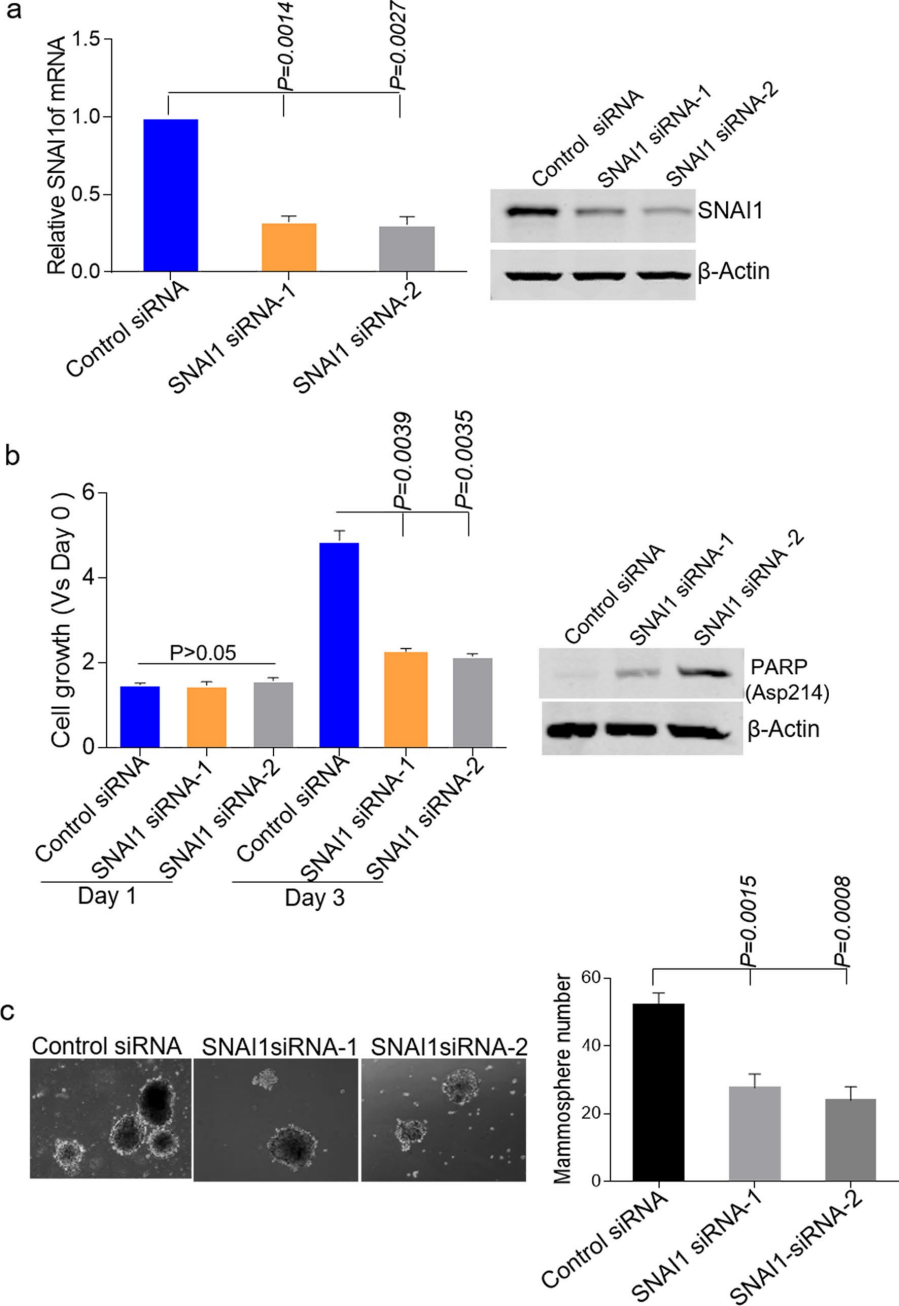
Knockdown of Snail sensitizes TN-resistant HCC1806 cells to triptonide. (**a**) RT-qPCR (left) and western blot (right) analysis of SNAI knockdown in TN-resistant HCC1806 cells transfected with control siRNA, SNAI1 siRNA-1, or SNAI1 siRNA-2 for 48 h. Each bar is presented as mean ± SD (n = 3) (***, p < 0.001). (**b**) Cell growth assays using TN-resistant HCC1806 cells pre-transfected with control or SNAI1 siRNAs. Cells were grown in the presence of triptonide. Each bar is presented as mean ± SD (n = 3) (p > 0.05, **, p < 0.01). Western blot analysis of cleaved PARP levels in TN-resistant HCC1806 cells transfected with control or SNAI1 siRNAs. (**c**) Mammosphere formation assays in control and SNAI1-knockdown TN-resistant HCC1806 cells. Each bar is presented as mean ± SD (n = 3) (**, p < 0.01). Magnification, × 200.

To investigate whether increased SNAI1 contributes to resistance to triptonide, we knocked down SNAI1 expression in TN-resistant HCC1806 cells using two different SNAI1 siRNAs, as shown by qRT-PCR and immunoblotting analysis (Fig. 4a). These cells were treated with SNAI1 siRNA-1 or siRNA-2 for 48 h, followed by triptonide treatment for 24 or 72 h. Cell proliferation assays showed that SNAI1 knockdown by siRNA-1 or -2 rendered the TN-resistant cells susceptible to triptonide treatment (Fig. 4b, left). Immunoblotting showed that an increase of cleaved PARP levels was elicited by siRNA-mediated SNAI1 knockdown (Fig. 4b, right). Furthermore, SNAI1 knockdown in TN-resistant TNBC cells impaired mammosphere formation (Fig. 4c), which is consistent with the well-established role of SNAI1 in CSC function. Therefore, our data indicate that highly elevated SNAI1 expression is involved in acquired resistance to triptonide in TNBC cells.

## Discussion

In this study, we show that triptonide inhibits TNBC cell and xenograft tumor growth and attenuates CSC properties. We uncover that JNK-mediated SNAI1 induction represents a negative feedback response that may counteract the natural anti-cancer agent and is involved in acquired resistance to triptonide in TNBC cells. Our study may open up new avenues for exploring alternative and complementary medicine in the treatment of TNBC.

Importantly, our finding that triptonide has a potent growth-inhibitory effect in TNBC cells further helps establish the natural compound as a promising anti-cancer agent. Previous studies showed that triptonide induces cellular senescence and apoptosis by suppressing transcription of TERT and oncogenic c-MYC in acute myeloid leukemia cells^9^. Our work also indicate that Myc is also suppressed by triptonide in TNBC cells. Notably, Zhang, M. et al*.* found that triptonide inhibits lung cancer cell growth, stemness, and tumorigenicity via blocking Gli signaling^17^. This is consistent with our results of triptonide effects on TNBC CSCs and stem cell-associated gene expression.

Currently, little is known about the regulation of cancer cell response and resistance to triptonide. We discovered that SNAI1 is upregulated by triptonide, albeit moderately. It is well-established that SNAI1 is a master regulator of EMT, CSC, and cell migration^18–20^. Its overexpression induces multi-drug resistance in breast cancer cells^21^. In line with the notion that anti-cancer therapy resistance-associated genes are commonly increased by anti-cancer drugs as a feedback response, we further showed that SNAI1 expression is drastically elevated in in vitro derived TN-resistant TNBC cells. Knockdown of SNAI1 in these cells restored sensitivity to triptonide. Our study may represent the first report showing in vitro models and mechanisms for potential resistance to triptonide treatment in cancer cells, suggesting that targeting SANI1 pathways may overcome the acquired resistance.

Another novel finding of our study is the regulation of SNAI1 expression by JNK activation, a common signal elicited by stress conditions. Previous studies have established AKT and ERK as important inducers of SNAI1 transcription in cancer cells. Surprisingly, they are not involved in the increase of SNAI1 expression in triptonide-treated and -resistant TNBC cells, whereas JNK was found to be an activator of SNAI1 expression. This result provides a poorly characterized link between JNK signaling and SNAI1. The biochemical mechanism mediating the JNK regulation of SNAI1 expression remains to be elucidated.

It is noted that KLF17 is upregulated while Id1 is downregulated by TN treatment in TNBC cells. Notably, KLF17 levels are lower in TN-resistant TNBC cells than in parental cells. KLF17 is a negative regulator of EMT and functions as a tumor suppressor. Low expression of KLF17 is associated with cancer progression^22^. A previous report showed that KLF17 directly suppresses the transcription of *Id1*^13^, a critical transcription factor in EMT and metastasis^23^. Whether KLF17 suppresses SNAI1 expression and mediates the effects of triptonide on SNAI awaits further investigation.

In conclusion, our study demonstrates that triptolide may serve as a potential effective therapeutic agent to treat TNBC. Further understanding the regulatory mechanism of SNAI1 expression in TN-resistant TNBC cells may help develop strategies to improve the effectiveness of triptonide-based alternative and complementary therapy. This study warrants further in-depth investigation of the utility of triptonide and similar natural products in TNBC treatment.

## Materials and methods

### Reagents

All chemicals were purchased from Sigma-Aldrich (St. Louis, MO, USA) unless otherwise stated. Dulbecco’s Modified Eagle Medium (DMEM) and DMEM/F12 were purchased from Thermo Scientific (Waltham, MA, USA). 0.25% Trypsin EDTA was from Corning (Houston, TX, USA). Penicillin–Streptomycin Solution was from Fisher (Houston, TX, USA).

### Cell culture

HCC1806, BT549, MDA-MB-231 were cultured in 5%CO2 at 37 °C in DMEM that contained 10% fetal bovine serum and 1% penicillin/streptomycin. Triptonide was diluted with dimethylsulfoxide (DMSO), and a 0.2 µM concentration was used for in vitro assays. TN-resistant HCC1806 cells were developed by culturing the parental cells in the presence of 0.2 µM concentrations of triptonide until cells show a similar growth rate in the absence or presence of triptonide.

### Quantitative RT-PCR

RNA was extracted using RNeasy Mini kit (Qiagen, Germany). then was reverse transcribed using QuantTect Reverse Transcription Kit (Qiagen, Valencia, CA). Transcript levels for targets related to Myc, ITGA6, KLF17, and SNAI1 were measured using the quantitative real-time iQ SYBR Green Supermix kit (Bio-Rad, Hercules, CA), and samples were normalized to endogenous β-Actin. Human primer sequences are ITGA6 (5′-GAGCTTTTGTGATGGGCGATT-3′, 5′-CTCTCCACCAACTTCATAAGGC-3′), Myc (5′-GAGCCCCTGGTGCTCCA-3′, 5′-GCAGAAGGTGATCCAGACTCTGA-3′), KLF17 (5′-GCCGTGGTGGCTGGTTC-3′, 5′-TTCTCGTTATCCTGGGCAGC-3′), SNAI1 (5′-CCTCCCTGTCAGATGAGGAC-3′, 5′-CCAGGCTGAGGTATTCCTTG-3′), β-Actin (5′-CCACCATGTACCCTGGCATT-3′, 5′-CGGACTCGTCATACTCCTGC-3′).

### Western blot analysis

Proteins were extracted using RIPA lysis buffer (ThermoFisher). Protein concentration was determined by the BCA Protein Assay Kit (Thermo Fisher, Waltham, MA). Proteins (40 µg) were run on 4–20% gradient gels and transferred onto PVDF membranes using a Trans-Blot Turbo transfer pack and Trans-Blo Turbo transfer system (Bio-Rad). Membranes were blocked with Odyssey blocking buffer (LI-COR) and incubated with primary antibodies overnight at 4 °C. The primary antibodies were SNAI1 (1:500, 3895 s, Cell signaling), KLF17 (1:500, sc-398132, Santa Cruz), ITGA6 (1:500, sc-374057, Santa Cruz), MYC (1:500, 2272 s, Cell signaling), Cleaved PARP (1:500, 5625 s, Cell signaling), β-Actin (A-9) (1:500, sc-1616, Santa Cruz). p-AKT (1:500, #9217c, Cell signaling), p-ERK (1:500,), p-JNK (1:500, sc-6254, Santa Cruz). total-JNK (1:500,), ERK inhibitor U0126 (10 µM, 19–147, Millipore), JNK inhibitor SP 600,125 (10 µM, S1460, Selleckchem).

### Cell viability assay

Cells were seeded into 96-well plates at a density of 2000 cells per well with parental groups and TN treatment group. All samples were run in triplicate. Cells were incubated for 24, 48, and 72 h followed by a cell viability assay using the Cell Titer-Glo luminescence assay (Promega) as per manufacturer’s instructions. Images were taken using an EVOS FL Auto microscope (Life Technologies, Carlsbad, CA).

### RT^2^ profiler PCR array

HCC1806 cells were treated with triptonide for 10 h. Total RNA was extracted and quantified by NanoDrop 2000 Spectrophotometer (NanoDrop, Technologies). First-strand cDNA was synthesized using the QuantTect Reverse Transcription Kit. The expression of the 84 genes was analyzed using the ΔΔC_T_ method of quantification with RT^2^ Profiler PCR Array kit (PAHS-176ZA, Qiagen, USA).

### Transient transfection

HCC1806 TN-resistant cells were transfected with 60 nM Control siRNA (sc-44230, Santa Cruz), 60 nM SNAI1 siRNA-1 (GCAAAUACUGCAACAAGGA, D-010847-01, Dharmacon, TX), 60 nM SNAI1 siRNA-2 (GCUCGGACCUUCUCCCCGAA, D-010847-03, Dharmacon, TX) for 48-h using Lipofectamine 2000 reagent (Invitrogen, Carlsbad, CA). Cells were then subjected to SNAI1gene expression analysis, cell viability assays, and mammosphere formation assays.

### Mammosphere formation assay

One thousand single cells were plated into 6-well ultra-low attachment plates (Corning), MammoCult Human Medium (StemCell Technologies) supplemented with Heparin (4 μg/ml, StemCellTechnologies) and Hydrocortisone (0.48 μg/ml, StemCell Technologies) was used. Fresh medium was added every two days. Triptonide was added after culture one week. Mammospheres were counted after 96 h of triptonide treatment.

Images were taken using an EVOS FL Auto microscope (Life Technologies, Carlsbad, CA).

### ALDH activity assay

Aldehyde Dehydrogenase Activity Colorimetric Assay (cat# MAK082, Sigma-Aldrich) was used to measure ALDH activity. HCC1806 cells were treated by 0.2 µM triptonide for 20 h. Then 1 × 10^6^ cells were homogenized in 200 µL of ice-cold ALDH assay buffer. Aliquots of 5 µL,10 µL, 25 µL, and 50 µL were added to the reaction mixture with a final volume of 50 µL. The mixture was incubated for 5 min and absorbance at 450 nm was measured with Promega ELISA program. Then the measurement was repeated every 2–3 min until the reading was greater than the value of the highest standard. The calculation was followed measurement method (ALDH activity = B * Sample dilution factor / (reaction time * V), where B = amount of NADH generated between Tinitial and Tfinal, Reaction time = Tinitial -Tfinal (minutes), V = Sample volume (mL) added to well. All samples and standards run in duplicate.

### In vivo experiments

Animal studies were conducted with the approval of the Shantou University Medical College Animal Care and Use Committee in accordance with the National Institutes of Health guidelines for the Care and Use of Laboratory Animals. The studies were in compliance with the ARRIVE guidelines. A total of 1 × 10^6^ MDA-MB-231 cells in 200 µL PBS was inoculated subcutaneously into the No. 4 mammary glands of 6-week-old female nude mice. Tumors were measured twice every week with calipers. Tumor volumes were calculated by the following formula: *a*^2^ × *b* × *0.5,* where *a* is the smallest diameter and *b* is the diameter perpendicular to a. Triptonide was dissolved in DMSO and kept at -20̊ C. When the tumors grew to ∼150 mm^3^, the mice were randomly divided into 2 groups. Mice in each group were treated once daily by oral gavage with triptonide (5 mg/kg) or the same amount of vehicle for 17 days. Tumor xenografts were immediately removed after cessation of drug treatment. The body weight, feeding behavior, and motor activity of each animal were monitored daily for general health evaluation. Isolated tumor tissues were analyzed by immunohistochemistry (cleaved PARP and Ki67) and the terminal deoxynucleotidyl transferase dUTP nick end labeling (TUNEL) assay.

### Annexin V apoptosis assay

HCC1806 cells were treated by 0.2 µM triptonide for 20 h. Approximately 2 × 10^5^ cells were suspended in 1 × annexin-binding buffer. After FITC annexin V and PI were added, the mixture was incubated at room temperature for 15 min. Apoptotic cells were detected using the FITC Annexin V/Dead Cell Apoptosis kit (#V13242, Invitrogen) according to the manufacturer’s instruction. Samples were analyzed on the BD LSR Fortessa Flow Cytometer (BD Biosciences). Cell debris was excluded in SSC-A VS FSC-A plot. Single cell populations were gated from SSC-W VS SSC-H plot. More than 10,000 single cells were recorded from each sample. Data was analyzed using BD FACSDiva V8.02 software.

### Statistical analysis

All experiments were performed three times with samples measured in duplicate or triplicate. Results are expressed as mean ± standard deviation (SD), unless otherwise stated. GraphPad Prism 6.0 software (GraphPad Software, San Diego, CA) was used for statistical analysis. P < 0.05 was considered statistically significant.

## Supplementary Information

Supplementary Information.
