# Supplementary material for: Identification of triptonide as a therapeutic agent for triple negative breast cancer treatment
Source: Sci Rep. 2021 Jan 28;11:2408. doi: 10.1038/s41598-021-82128-0 (PMC7843598; doi:10.1038/s41598-021-82128-0)

Supplementary Information

Title: Identification of Triptonide as a Therapeutic Agent for Triple Negative Breast Cancer Treatment

Authors: Bowen Gao, Jiongyu Chen, Bingchen Han, Xinfeng Zhang, Jijun Hao, Armando E. Giuliano, Yukun Cui, Xiaojiang Cui

Supplementary Figure Legends

Figure S1. Dose-response studies of the triptonide effect on cell proliferation.

(a) HCC1806 cells were treated 24 hours with triptonide at different concentrations (0.01µM, 0.1 µM, 0.2 µM, 0.5 µM, and 1 µM) using the Cell Titer-Glo luminescence assay. Each experiment was repeated twice. Each bar is presented as mean ± SD (n = 3). (b) representative images showing the effects of 0.2 µM triptonide treatment (24 hours) in ER+ MCF-7 and HER2+ SKBR3 cells. Magnification, x 200. Right, the effect of 0.2 µM triptonide on cell growth measured by CellTiter-Glo assays. MCF-7 and SKBR3 cells were treated for 24 (D1), 48 (D2), and 72 hours (D3). Each bar is presented as mean ± SD (n = 3). (c) Examination of cell apoptosis in fixed xenograft tumor tissues by TUNEL assays (×400 magnification). Images show induced apoptosis in triptonide-treated tumor tissues.

Figure S2. Triptonide attenuates stem-like properties of TNBC cells.

(a) List of genes with 4-fold or more changes in expression levels detected by RT^2 profiler PCR analysis in HCC1806 cells. (b) Examination of ITGA6, Myc, KLF17, and SNAI1 expression by qRT-PCR in MDA-MB-231 TNBC cells. Each bar is presented as mean ± SD (n = 3) (*, $p < 0.05$. **, $p < 0.01$. ***, $p < 0.001$).

Figure S3. SNAI1 expression is regulated by JNK activation in TNBC cells.

The effects of ERK inhibition by 10 µM U0126 and JNK inhibition by 10 µM SP600126 on SNAI1 expression in HCC1806 cells were examined by immunoblotting.

Figure S1

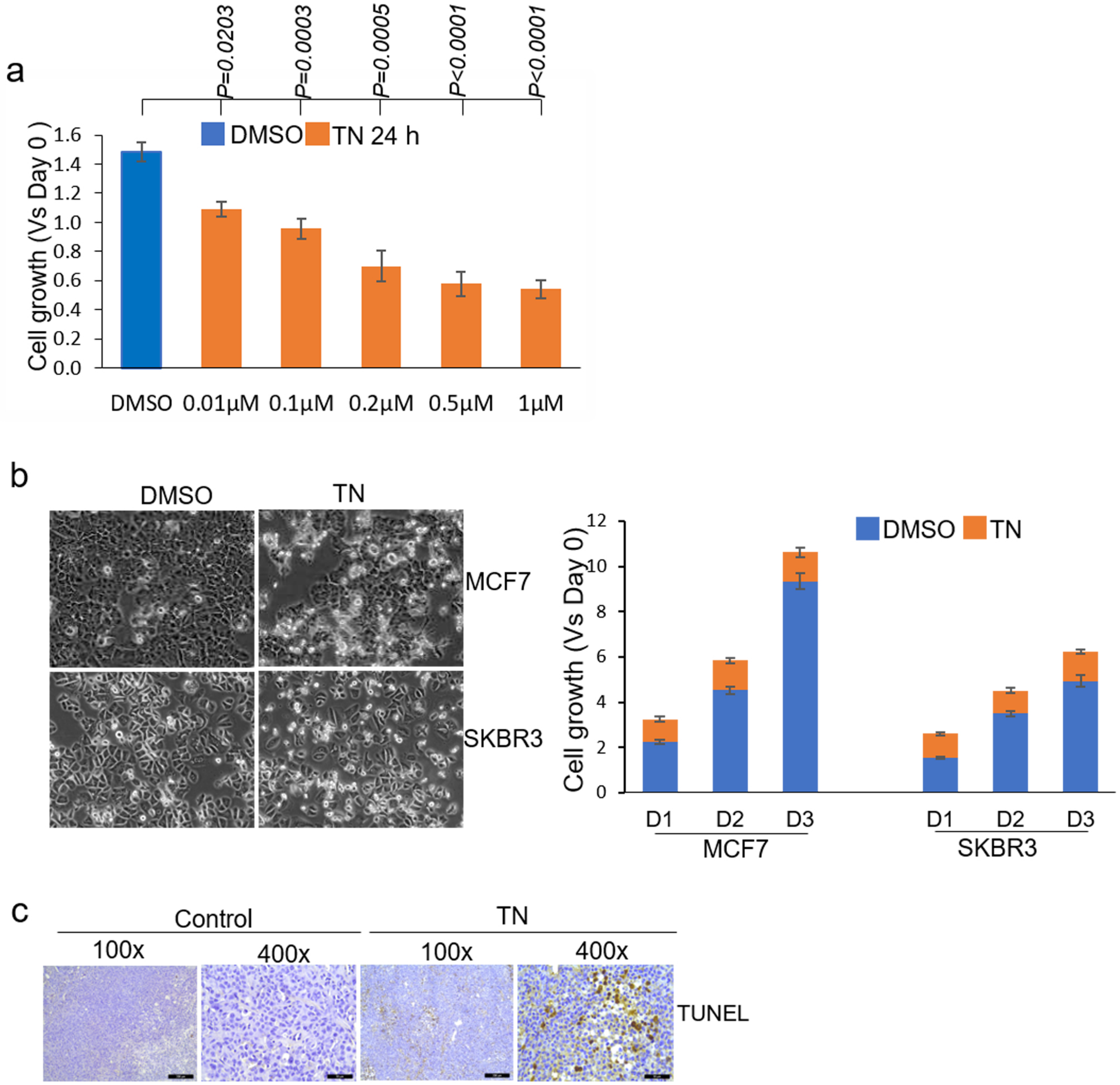

a
P=0.0203
P=0.0003
P=0.0005
P<0.0001
P<0.0001
DMSO
TN 24 h
Cell growth (Vs Day 0)
1.6
1.4
1.2
1.0
0.8
0.6
0.4
0.2
0.0
DMSO 0.01μM 0.1μM 0.2μM 0.5μM 1μM
b
DMSO
TN
MCF7
SKBR3
DMSO
TN
Cell growth (Vs Day 0)
12
10
8
6
4
2
0
D1 D2 D3
MCF7
D1 D2 D3
SKBR3
c
Control
TN
100x
400x
100x
400x
TUNEL

Figure S2

a

Position	Gene Symbol	Fold Regulation	p-Value
G06	**TWIST**2	398.51	N/A
F11	**SNAI1**	29.36	N/A
D09	**KLF17**	13.05	N/A
E07	MYCN	9.66	N/A
E10	NOS2	4.71	N/A
A11	CD34	4.30	N/A

Position	Gene Symbol	Fold Regulation	p-Value
D03	**ITGA**6	-1992.17	N/A
C10	ID1	-22.12	N/A
B05	DKK1	-14.90	N/A
E06	**MYC**	-14.69	N/A
D07	KIT	-11.96	N/A
C12	CXCL8	-9.23	N/A
D08	KITLG	-7.25	N/A
A06	ATXN1	-5.33	N/A
D06	JAK2	-4.99	N/A
H06	HGDC	-4.84	N/A
E11	NOTCH1	-4.71	N/A
G12	ZEB2	-4.69	N/A
G07	WEE1	-4.25	N/A
C02	FGFR2	-4.25	N/A

b

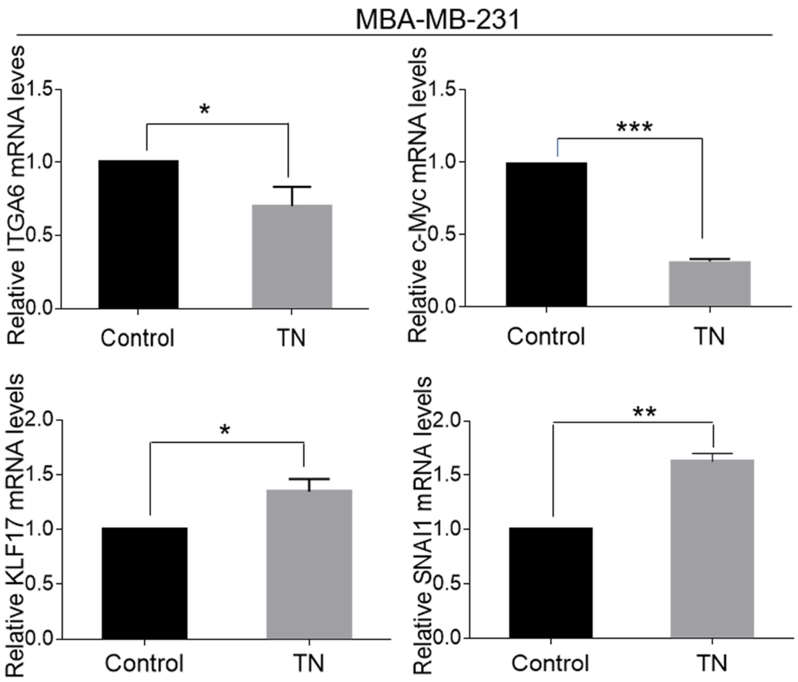

Figure S3

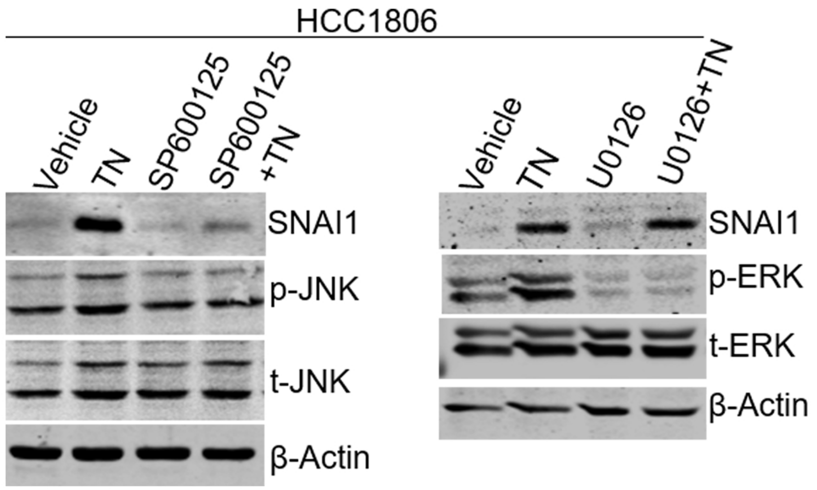
HCC1806
Vehicle
TN
SP600125
SP600125 +TN
Vehicle
TN
U0126
U0126+TN
SNAI1
p-JNK
t-JNK
β-Actin
SNAI1
p-ERK
t-ERK
β-Actin

Figure S4, full-length images

Figure 1c

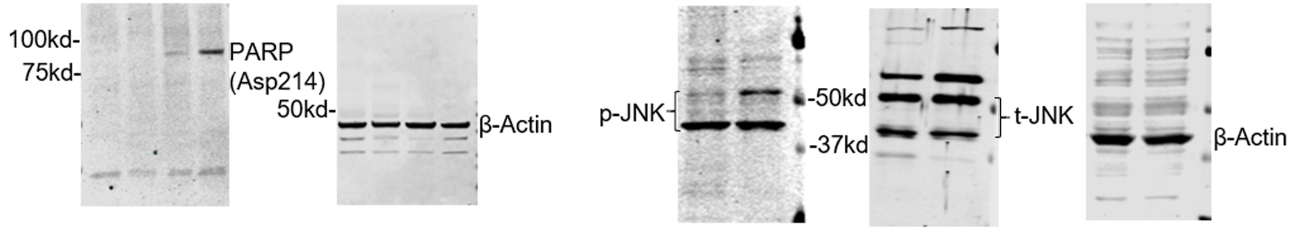

Figure 2e

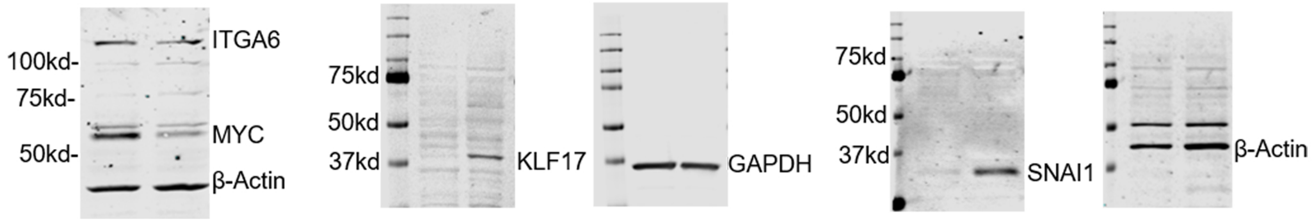

Figure 3c

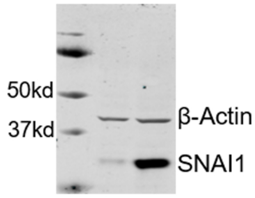

Figure 3d

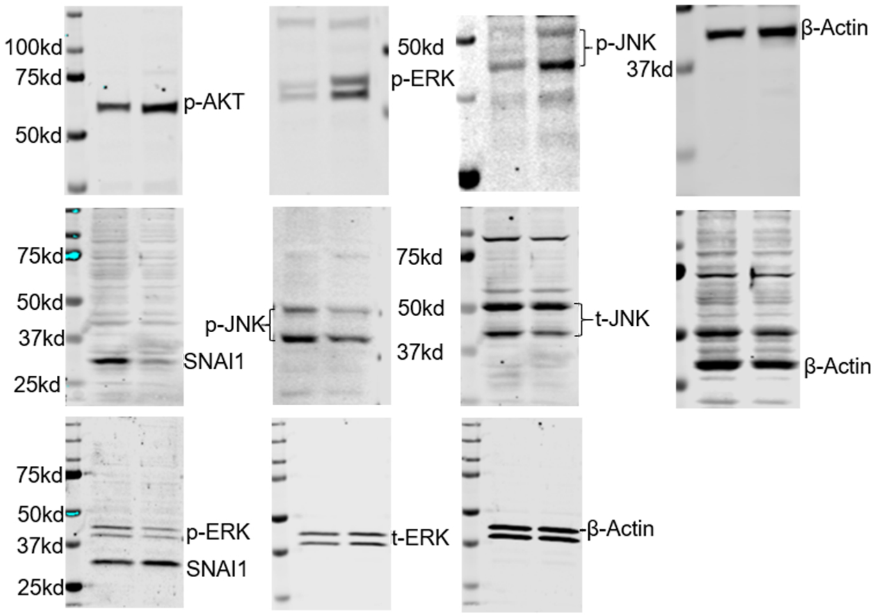

Figure 4a

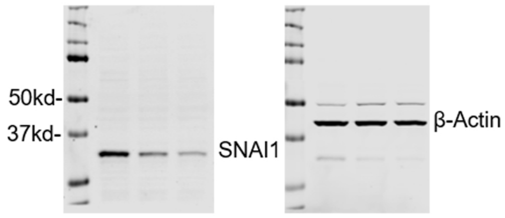

Figure 4b

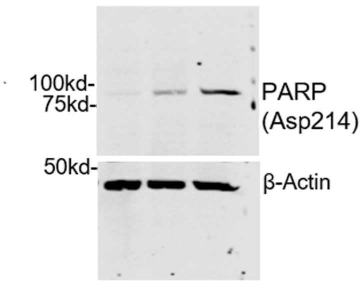

Figure S5, full-length images

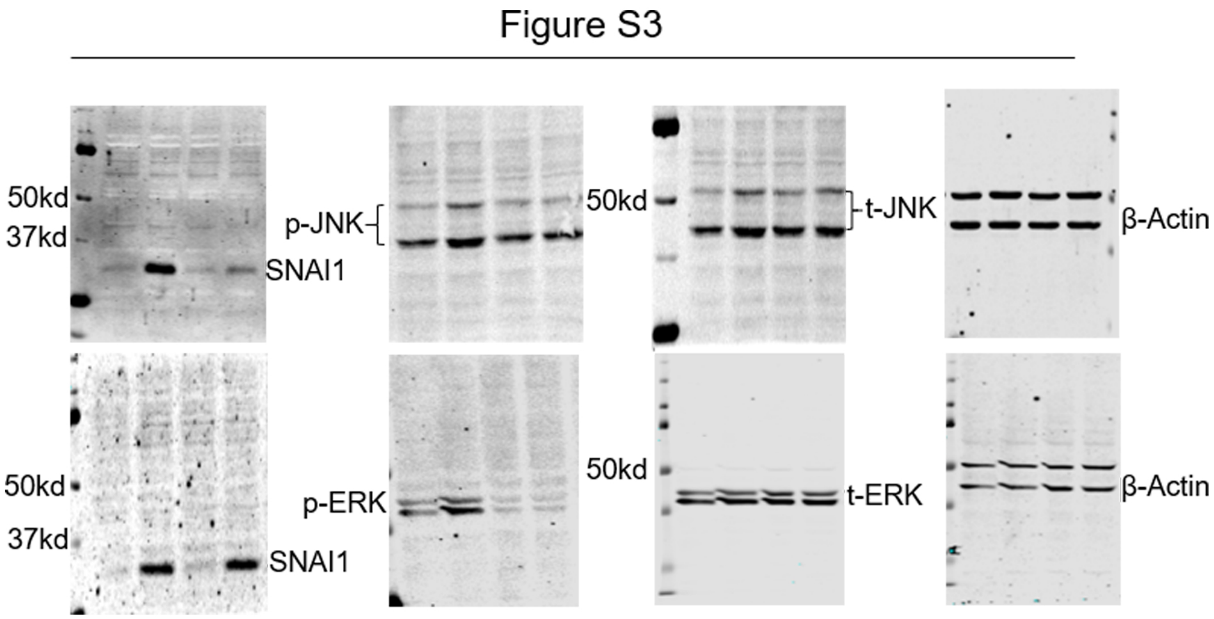

Supplement: Supplementary file 1 — Supplementary Information. [file 41598_2021_82128_MOESM1_ESM.pdf]
